# Homovanillic acid and vanillylmandelic acid as neuroblastoma biomarkers in children

**DOI:** 10.11613/BM.2026.020505

**Published:** 2026-06-15

**Authors:** Sina Kavalakatt, Josko Ivica

**Affiliations:** 1 Custom Biologics, Toronto, Canada; 2 McMaster University, Department of Pathology and Molecular Medicine, Hamilton, Canada

**Keywords:** homovanillic acid, neuroblastoma, reference intervals, vanillylmandelic acid

## Abstract

Neuroblastoma (NBL) is the most common pediatric tumor with the highest fatality rate. The survival rate is improved when NBL is diagnosed in children under 1 year of age, but the diagnosis is complex due to the heterogeneity of the disease. This tumor type is characterized by elevated catecholamines and their metabolites, particularly homovanillic acid (HVA) and vanillylmandelic acid (VMA). Numerous analytical methods measuring urinary HVA and VMA have been developed to support the early NBL diagnosis. However, it is difficult to establish age-related reference ranges for these tests in children. To support the diagnostic process, the aim of this review is to provide up-to-date published age-related HVA and VMA reference intervals with focus on neuroblastoma development, catecholamine metabolism and possible interferences in the analytical methods used.

## Graphical abstract

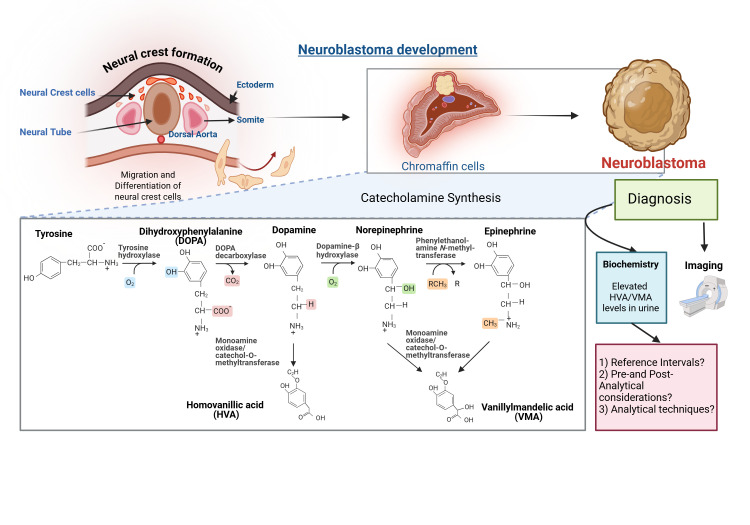

## Highlights

• Neuroblastoma is a fatal tumor diagnosed in children

• It is characterized by high concentration of urine homovanillic and vanillylmandelic acid

• Although diagnosis is complex, early diagnosis is crucial for survival rate

• Various analytical methods have been developed, such as HPLC-ECD and LC-MS/MS

• Laboratories performing these tests should establish their own reference intervals

## Introduction

Neuroblastoma (NBL) is the most common extracranial solid tumor in the pediatric population with a 5-year survival rate of 50% (*1*). Neuroblastoma accounts for 8% of pediatric cancer and contributes to 15% of cancer-related deaths in children. The disease has a highly diverse prognosis depending on the location of the tumor and age at diagnosis (*1*). If diagnosed when the child is less than a year old, the 5-year survival rate is significantly higher compared to those who were diagnosed at greater than one year of age. Early detection is possible through screening of biomarkers excreted in detectable quantities.

Neuroblastoma originates in the neural crest cells and is commonly located in the adrenal medulla or sympathetic chain. The tumor can arise anywhere from the abdomen, regional lymph nodes, bone marrow, cortex, and liver. Uncommonly, obstruction of facial and ophthalmic veins can occur due to the tumor leading to an appearance of “raccoon eyes” (*2*). Furthermore, disparities in risk and survival rates depend on race and ethnicity in the pediatric population (*3*). Genetic differences were thought to be one of the main causes for the higher prevalence of high-risk NBL in black and Native American subjects (*3*).

This neuroendocrine tumor is the most common catecholamine-producing tumor of the sympathetic nervous tissue (*4*). The major catecholamines are noradrenaline (nor-epinephrine), adrenaline (epinephrine), and dopamine, with the latter being most elevated in NBL. Most catecholamines produced by neuroblastoma cells are metabolized to vanillylmandelic acid (VMA) by hepatic alcohol dehydrogenase. Dopamine is metabolized completely to homovanillic acid (HVA). These catecholamine metabolites are excreted into urine in relatively large amounts with micromolar concentrations allowing ease of measurement (*5*).

The recommended criteria for diagnosis of NBL are either unequivocal pathologic diagnosis from tumor tissue with/without immunohistology, electron microscopy, increased urine or serum catecholamines or metabolites; or bone marrow aspirate biopsy containing unequivocal tumor cells and increased urine or serum catecholamines or metabolites. Homovanillic acid and VMA have served as valuable clinical biomarkers since the 1970s for diagnosing and follow up NBL (*6*, *7*).

The combined biochemical detection of urinary HVA and VMA allows NBL diagnosis with high sensitivity (66-100%) and high specificity (> 99%) (*8*). In Vietnamese children, aged less than 6 months up to 15 years, sensitivity of random spot urine HVA was 88% and 82% for VMA, meaning that 12% and 18% will have falsely negative HVA and VMA, respectively, even though these children had NBL. Specificity for both random spot urine HVA and VMA was similar (98% *vs.* 97%), meaning that only 2% and 3% were falsely negative for HVA and VMA, respectively. Positive (LR+) and negative likelihood (LR-) ratio for a random spot urine HVA was 39.85 and 0.12, respectively, while LR+ and LR- for a random spot urine VMA was 37.1 and 0.18, respectively (*9*). In another study from France, the authors concluded that the specificity and sensitivity for spot urine HVA at the cut-off of 23 nmol/µmol creatinine was 99% and 82%, respectively, with the area under the curve (AUC) of 0.90. Meanwhile, the specificity and sensitivity for VMA, at the cut-off of 12 nmol/µmol creatinine, was 95% and 91%, respectively, with the AUC of 0.936. This diagnostic performance was seen in children aged less than 1 year of age. The authors also did test accuracy in children aged between 1-5 years of age. In this group, the specificity and sensitivity for HVA at the cut-off of 13 nmol/µmol creatinine was 99% and 100%, respectively, with the AUC of 1.00. For the same age group, VMA at the cut-off of 6 nmol/µmol creatinine the specificity was slightly lower at 97% with the same sensitivity and the overall AUC being 0.99. The oldest age group, 5-10 years of age, had both specificity and sensitivity for HVA at the cut-off of 19 nmol/µmol creatinine 100% with AUC of 1.00, while VMA at the cut-off of 5 nmol/µmol creatinine specificity was lower with 96% and the same sensitivity as HVA (*i.e.* 100%). The AUC for VMA in this age group, *i.e.* 5-10 years old, was 0.991 (*10*).

Homovanillic acid and VMA have similar characteristics making it convenient to quantitate both analytes from the same specimen. Different specimens have been used for quantification including 24-hour urine and spot urine, which always must be corrected or normalized for creatinine excretion (HVA/Cr and VMA/Cr). The standard practice requires the use of urine samples obtained from 24-hour collections to account for the effect of the circadian cycle variations in metabolite secretion. Studies have demonstrated the interchangeability between samples from 24-hour collections and single spot urine (*11*). The difficulty of obtaining a 24-hour collection from a young child, studies supporting that the variations in excretion of HVA and VMA during different times of the day is due to renal excretion variation, and the ease of spot urine collection increases the popularity of spot urine samples (*12*). The detection of NBL patients with positive HVA and/or VMA depends on the stage of the disease with abnormal concentration at later stages of the tumor (*13*). In this review, we have explored the published reference interval data for urinary HVA and VMA in children from birth to 19 years of age, the analytical methods used for these analytes, preanalytical considerations, and possible analytical interferences with special attention to main pathway of NBL development and catecholamine metabolism.

## Neuroblastoma development and molecular pathogenesis

The cells of origin are mostly from the sympathoadrenal cells of the neural crest that differentiate into catecholamine-secreting chromaffin cells and ganglion cells (*14*). Several biomarkers have been assessed to enable accurate diagnosis and follow-up for NBL. Disialoganglioside (GD2) is one such well investigated surface antigen, abundantly expressed in the outer membrane of all neuroblastomas. Disialoganglioside has been suggested to be integral in the formation and maintenance of membrane microdomains in neural tissues. Histological detection of GD2 can be used to detect NBL; however, the promising utilization has been as an antigen for immunotherapy (*15*). The receptor tyrosine kinases, TrkA, TrkB and TrkC are neurotrophin receptors that are critical for development and maintenance of the peripheral nervous system. While elevated expression of *TrkA* indicates low-grade NBL to spontaneous regression or differentiation, high expression of *TrkB* has been correlated with high-risk NBL and low patient survival rates (*16*).

**Figure 1:**
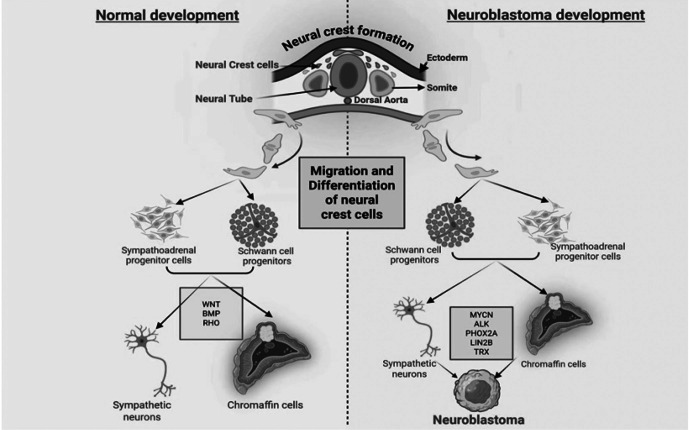
Development of neuroblastoma from neural crest cells. Neural crest cells undergo epithelial to mesenchymal transition, delaminating, migrating and differentiating to somites or towards dorsal aorta. Dorsal aorta neural crest cells start to differentiate into sympathoadrenal progenitor cells or Schwann cell progenitors, eventually giving rise to sympathetic neurons or chromaffin cells respectively. This process is regulated by complex signaling pathways. Dysregulation of factors such as MYCN, ALK, PHOX2A, LIN2B and TRX, induce changes in cell specification, migration and cell differentiation resulting in hyperneoplastic lesions resulting in neuroblastoma.

Elevated expression of Myelocytomatosis oncogene neuroblastoma-derived homolog gene (*MycN*) is integral for ventral migration and cell expansion within neural crest during normal sympathoadrenal development in murine models (Figure 1). It has been suggested that sympathoadrenal maturation happens independent of *MycN* expression. The sympathoadrenal precursor cells matures into neural or chromaffin cells. In absence of signals or response to signals determining neuronal and chromaffin cell fate, preneoplastic lesions in sympathoadrenal precursor cells can develop into NBL (*17*). Controlled apoptosis occurs in the maturation stages to circumvent effects of the excess precursor cells produced. The persistent high concentration of *MycN*, a master transcription factor, in the maturation phase could result in sustained proliferation and eventual development of NBL (*18*).

Other common genetic markers studied are the *MYCN* gene amplification, anaplastic lymphoma kinase (*ALK*) mutations, paired-like homeobox 2B (*PHO2XB*) loss of function, mutations, and inactivating transcriptional regulator *ATRX* mutations (*19*). An accumulation of these mutations also adds further complexity to the pathogenesis of the disease. Although numerous markers have been studied, no single marker has been able to provide an accurate diagnosis. A combination of imaging, cell surface markers, genetic analysis and measurement of secreted catecholamine metabolites are used for diagnosis and monitoring of NBL treatments in patients.

## Vanillylmandelic and homovanillic acid metabolism and secretion

Neuroblastoma is characterized by the synthesis, metabolism, and secretion of catecholamines and related metabolites - HVA and VMA. The pathways involved in biosynthesis, metabolism, storage, and secretion of these biomarkers highlight their diagnostic value.

The biosynthesis of catecholamines starts with the conversion of tyrosine, by tyrosine hydroxylase to 3,4-dihydroxyphenylalanine (DOPA). Aromatic- L-amino acid decarboxylase converts DOPA to dopamine, which is then transported into storage granules by vesicular monoamine transporters. Dopamine is further converted to norepinephrine by dopamine β-hydroxylase, an enzyme exclusively present in the storage granules. Norepinephrine is converted to epinephrine depending on the presence of phenylethanolamine N-methyltransferase (PNMT) in the adrenal chromaffin cells (*20*). This enzyme is present in the cytosol; hence this conversion depends on the transfer of norepinephrine from the storage granules to the cytoplasm. Epinephrine is then translocated back into the storage granule and actively secreted as a hormone.

**Figure 2:**
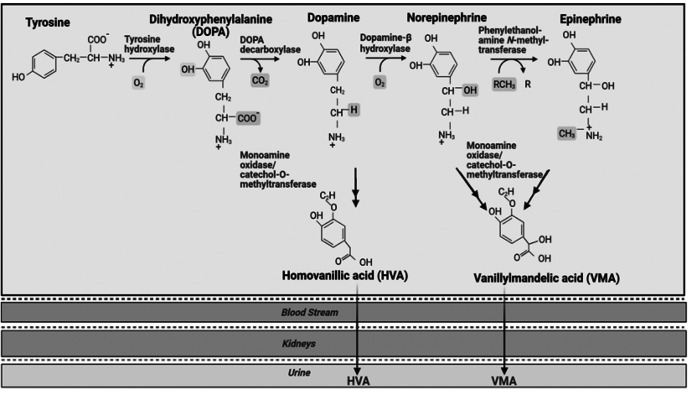
Catecholamine metabolism and secretion. Initial biosynthesis of dopamine, norepinephrine and epinephrine occur in chromaffin cells of the adrenal medulla. Catecholamines are synthesized from tyrosine derived from the food consumed or phenylalanine in the liver. Tyrosine conversion to dihydroxyphenylalanine (DOPA) is catalyzed by tyrosine hydroxylase. Enzyme DOPA decarboxylase converts DOPA to dopamine. Dopamine is converted to noradrenaline by hydroxylation of the dopamine side chain catalyzed by dopamine-β-hydroxylase. Noradrenaline is converted to adrenaline by phenylethanolamine N-methyltransferase (PNMT). 90% of the catecholamines are taken up by the nerve endings after which it is utilized or deaminated by monoamine oxidase (MAO). Monoamine oxidase and catechol-O-methyltransferase (COMT) are responsible for converting dopamine and norepinephrine/epinephrine to homovanillic acid (HVA) and vanillylmandelic acid (VMA). The main metabolic pathways take place in different compartments followed by entry into the bloodstream, clearance and metabolism by kidneys for elimination in urine.

The secretion of catecholamines is initiated by exocytotic secretion of the vesicular stores into the surrounding extracellular space. The secretion of norepinephrine from sympathetic neurons occurs locally and neuronal uptake takes place releasing only a small proportion into the bloodstream (*20*). 3,4-dihydroxyphenylglycol (DHPG) is converted by monoamine oxidase (MAO) from norepinephrine. Further metabolism of DHPG is performed by catechol-O-methyltransferase (COMT) to 3-methoxy-4-hydroxyphenylglycol (MHPG) and most MHPG is converted to VMA in the liver by alcohol dehydrogenase (*21*). Dopamine is metabolized to HVA by the combined actions of COMT and MAO. The pathways are detailed in Figure 2. LaBrosse *et al.* showed that neuroblastomas lacked catecholamine storage vesicles, a characteristic of mature chromaffin cells (*22*). As these tumors do not generally present with hypertension or with varied urinary catecholamine metabolites, hence measurement of catecholamine metabolites particularly HVA and VMA have been used for diagnosis.

## Sample pretreatment and preanalytical considerations

In individuals without NBL, activation of the sympathetic nervous system is a factor responsible for higher concentrations of catecholamine metabolites. Other confounding factors including acute illness, diet, exercise, medication, patient posture during sampling, accuracy of sampling time and sample stability have been shown to influence catecholamine and related metabolite concentrations (*23*).

## Effect of diet and exercise

Ingestion of dopamine rich foods such as bananas, pineapples, nuts, beans and tomatoes have been suggested to impact the concentrations of catecholamine and their metabolites in urine samples (*24*). The enzymatic activity of sulphotransferase enzyme (SULT1A3), is known to influence the impact of dietary catecholamines in urine. Sulphotransferase found in the gastrointestinal tract, liver, platelets and nervous tissue, rapidly sulfates dietary catecholamines before systemic absorption. A reduced or variable SULT1A3 activity can result in increased circulating catecholamines thereby increasing the risk of false positive results in urinary catecholamine as well as HVA/VMA testing. Hence, although avoiding catecholamine rich food is recommended, an overnight fast can be a solution to circumvent the dietary effects.

Increased HVA/VMA secretion has been studied in cases of acute illness; hence such an episode requires documentation and confirmation from the patient. Supine position of the patient has been shown to significantly reduce catecholamine secretion and consequently urinary HVA and VMA concentration in comparison to sample collection in seated position (*25*). Physical activity has been correlated to increased HVA/VMA concentrations; hence it has been recommended to avoid intense exercise before sample collection. Another confounding factor for high concentration of metanephrine metabolites, *i.e.* HVA and VMA, is renal failure. Pamporaki *et al.* have reported a 50% increase in metanephrine metabolites in patients requiring dialysis (*26*).

## Effect of medication

A common interfering factor in test interpretation is the interference of various medications in the analytical method used and pharmacological effects. While paracetamol and sulfasalazine have been reported to interfere with high-performance liquid chromatography coupled with electrochemical detection (HPLC-ECD), 3-O-methyldopa and midodrine interfere with liquid chromatography-tandem mass spectrometry (LC-MS/MS) methods. Various medications have been implicated in false results and are summarized in Table 1.

**Table 1:** Medication interference and type of effects

**Medications** **(reference)**	**Type of effect**	**Impact**
Atypical antipsychotics (Quetiapine, clozapine, risperidone) (*27*)	Pharmacophysiological	Clozapine and Quetiapine block α2-adrenoreceptors and Risperidone blocks D2 and serotonin 5-HT2A receptors; altering dopamine pathways and increasing VMA secretion
Monoamine oxidase inhibitors (*28*)	Pharmacophysiological	Inhibits oxidative deamination of catecholamines impairing HVA and VMA metabolism
α-adrenoreceptor blockers (doxazosin) (*29*)	Pharmacophysiological	α- adrenoreceptor antagonism; possible norepinephrine increase
Phenoxybenzamine (*30*)	Pharmacophysiological	α- adrenoreceptor antagonism; Increases norepinephrine/ normetanephrine concentration
Tricyclic antidepressants (Venlafaxine) (*30*)	Pharmacophysiological	Increases norepinephrine/ normetanephrine concentration; reduced norepinephrine neuronal uptake
3-O-methydopa (*23*)	LC-MS/MS	Interferes with L-dopa and dopamine metabolic pathway; decreases HVA and VMA concentration
Paracetamol (*31*)	Analytical interference	Interferes with oxidation/ colorimetric reactions in HPLC-ECD measurements
Amoxicillin (*32*)	Analytical interference	Co-elutes with urinary normetanephrine resulting in false positive HPLC-ECD results
Sulfasalazine (*33*)	Analytical interference	Interferes with current affecting accurate HPLC-ECD measurements
Labetalol (*30*)	Analytical interference	Labetalol metabolites could produce false signals leading to spurious elevation of HVA/VMA concentrations in HPLC-ECD
VMA - vanillylmandelic acid. HVA- homovanillic acid. HPLC-ECD - high performance liquid chromatography coupled with electrochemical detection. LC-MS/MS - liquid chromatography tandem mass spectrometry.		

Significantly elevated concentration of metanephrine metabolites have been reported due to pharmacological interferences. Medications such as tricyclic antidepressants and serotonin and norepinephrine reuptake inhibitors that reduce catecholamine uptake result in significantly increased VMA *via* increasing normetanephrine concentration. β- Blockers such as propranolol and atenolol have been suggested to be correlated with increased false positive results (*34*). Sympathomimetics and Parkinson’s medication L-Dopa may falsely increase HVA and VMA through stimulation of catecholamine production and consequently increased production of their end products *i.e.* HVA and VMA. On the other hand, monoamine oxidase inhibitors can cause decreased concentration of both HVA and VMA through inhibition of MAO, which in turn inhibits production of their precursors – catecholamines (*23*).

It is of prime importance that clinicians are aware of these interferences to avoid potential misdiagnosis of patients and suggest further testing in case of possible false positives.

## Sample collection and patient preparation

Twenty-four-hour urine or random urine, normalized by urine creatinine, collections are usually used for HVA/VMA measurement. However, the recommended specimen is 24-hour urine since NBL may not secrete the hormone (epinephrine and norepinephrine for VMA and dopamine for HVA) metabolites constantly over a day. The impact of diet and medications on HVA/VMA concentrations should be communicated to the patient. The patients should avoid intake of food that stimulates catecholamines synthesis and consequently HVA and/or VMA production, such as coffee, tea, especially green tea, bananas, citrus fruits, phenylethylamine from dark chocolate, cocoa and vanilla. It is also important that samples are collected under conditions of minimal physiological or emotional stress. The impact of acute illness and stressful situations on increased metanephrine and consequently increased VMA concentrations have been well studied (*35*). The reliability of the test is dependent on the timing of sample collection; hence the patient should ensure that all urine is collected in the 24-hour time frame. However, NBL is a pediatric cancer, and it is sometimes difficult to collect preferred specimens so for that reason clinical laboratories tend to accept random spot urine specimens. The quality of the urine collection can be improved by issuing clear instructions to clinicians and the patient prior to the collection.

Improved diagnostic performance was observed in urine samples collected at night due to the possible circadian effect resulting in lower excretion of catecholamines and consequently their end-products *i.e.* HVA and VMA (*36*).

## Analyte stability

Both HVA and VMA were assessed for their stability in the refrigerated conditions (between 2-8 °C) and pH between 1 and 4 with 6M HCl, 6M HNO_3_, or 50% acetic acid (*37*). The authors found that both analytes were stable for up to 7 days in these conditions (*38*). In another study, the authors performed the percent recoveries to assess the stability of HVA and VMA in native, acidified with HCl and citrate urines and found that both analytes were stable for up to 4 weeks at the ambient temperature, + 4 ºC, and - 20 ºC degrees (*39*). Another study, done on filter paper, assessed the stability of urinary HVA and VMA and concluded that both analytes were stable for up to 2 years frozen and refrigerated. On the other hand, both analytes were only stable for up to 3 weeks at room temperature (*40*).

## Analytical testing methods

The quantification of HVA and VMA has been performed using thin – layer chromatography (TLC), HPLC-ECD or coupled with fluorescence detection (FLD), gas chromatography-mass spectrometry (GC-MS), gas chromatography-flame ionization detector (GC-FID), enzyme immunoassay (EIA) and LC-MS/MS. Measurements have evolved over the past several decades with each methodology having advantages and disadvantages.

## Thin – layer chromatography

Historically, TLC was used to test for urinary HVA and VMA in confirmed cases of NBL. Takada *et al.* used TLC in ten Japanese children with established diagnosis of NBL, where both HVA and VMA were increased, and decreased after the cancer was either removed surgically or after chemotherapy treatment (*41*). In a paper by Takeda *et al.* the TLC method was also used for mass screening of neuroblastoma in Japanese children from 1981 until 1984 (*42*). Thin – layer chromatography was also used in investigation of circadian rhythms of HVA and VMA urinary excretion in healthy and depressed study participants (*43*).

## High-performance liquid chromatography

High-performance liquid chromatography coupled with ECD is the most commonly used technique for quantification of biogenic amines, not only HVA and VMA. Most amines are prone to oxidation with inherent fluorescence properties. Extensive sample preparation and a long time are required, although the design is straightforward enabling development and improvement of the method. In that regard, in the corresponding author’s laboratory urinary HVA and VMA were measured on HPLC-ECD. The method was extremely laborious with using solid phase extraction, using separate columns and mobile phases and was time consuming. Many published articles validated the analysis of not only HVA and/or VMA but also a few other tumor markers on HPLC-ECD (*44*-*46*). High-performance liquid chromatography coupled with FLD was also used in several publications, either to compare to a newer LC-MS/MS method, or to assess urine HVA and VMA, simultaneously with 5-hydroxyindoleacetic acid (5-HIAA) and 3, 4-dihydroxyphenylacetic acid (DOPAC) by HPLC-FLD (*47*, *48*).

## Gas chromatography

Owing to high identification, sensitivity, and separation power, GC-MS is an integral tool in clinical chemistry. Before the invention of mass spectrometry and its application with GC, GC-FID was used to measure various catecholamine metabolites (*49*). Lavreri *et al.* used GC-FID to confirm the analytical interference of the over-the-counter analgesic, antipyretic and anti-inflammatory medication ibuprofen. They determined that the ibuprofen metabolite, hydroxy-ibuprofen, interfered with urinary VMA (*50*). Beals *et al.* published a robust protocol for GC-MS method for simultaneous measurement of HVA and VMA (*51*). The Austrian neuroblastoma program developed the secondary stable isotope dilution GC-MS for HVA and VMA to help reduce unnecessary retesting of the primary HPLC method. Implementation of the GC-MS method reduced retesting from 11% (HPLC alone) to approximately 5% (*52*). In Vietnamese children, urinary HVA and VMA reference intervals (RI) were established by GC-MS. The authors optimized and validated a classic GC-MS method with derivatization (*9*). It has been used in the quantification of several amines including HVA and VMA. Although this technique allows the profiling of related compounds simultaneously, most analytes require extensive derivatization to become amenable to ionization. Consequent to low practicality, GC-MS application has been limited to only certain specialized endocrinology laboratories (*24*).

## Enzyme immunoassay

Enzyme immunoassay is based on the competitive reaction between catecholamine metabolites and corresponding monoclonal antibodies, labelled by HVA or VMA. Immunoassays enable full automation, high throughput and limited operator technical expertise. Thus, Yokomori *et al.* developed a mass screening of NBL in young Japanese infants. In their paper, they used two different monoclonal antibodies for HVA and VMA. The EIA measurement correlated well with the HPLC HVA and VMA measurement, providing the possibility of automation (*53*). Other countries that used EIA for HVA and VMA for the NBL screening were Austria and the state of Texas in the USA (*54*, *55*). In another study, a competitive EIA for urinary VMA was produced, where polyclonal antibody against VMA with a VMA-acetylcholinesterase labelled conjugate. Again, the EIA correlated well with HPLC method (*56*). Although immunoassays have the highest analytical sensitivity of all quantitation techniques, there are several limitations including specificity, accuracy, cross-reactivity and interference with other compounds. Further, different assays are required to assess related compounds. The high cost involved in developing the assay in-house limits the dependency on the diagnostic industry (*57*).

## Liquid chromatography-tandem mass spectrometry

Liquid chromatography-tandem mass spectrometry is a combination of separation capabilities of HPLC with high sensitivity, specificity and accuracy of mass spectrometric detection. It is the method of choice in several pharmaceutical and related industries for measurement of drugs. It is used to assess a wide range of compounds such as biogenic amines, steroids, thyroid hormones, thyroglobulin and vitamin D (*58*). Stable isotopes of analytes are used as internal controls to correct losses with sample pre-treatment, analyte separation and detection. Recently, LC-MS/MS has been replacing HPLC, GC-MS and immunoassay due to improved specificity, less runtimes and less intensive sample preparation. A difficulty with this technique is compound specific matrix effects (*59*). LC-MS/MS has been a method of choice since many clinical laboratories started acquiring this powerful but quite costly instrumentation. One article describes application of automation using solid liquid extraction for urinary HVA and VMA (*59*). Yu *et al.* described a development of a candidate reference measurement procedure for urinary HVA and VMA using the isotope dilution (ID) ID-LC-MS/MS (*60*). Many others developed and optimized an LC-MS/MS method in their laboratories, where this method replaced the older HPLC-ED method (*61*-*63*). Some other published methods also included 5-hydroxyindoleacetic acid (5-HIAA) to HVA and VMA (*64*, *65*). Another method by Xie *et al.* included urinary fractionated catecholamines (epinephrine, norepinephrine, dopamine), fractionated metanephrines (metanephrine, normetanephrine, 3-methoxythyramine), serotonin, 5-HIAA as well as HVA and VMA (*66*).

## Reference intervals

Reference intervals are considered as a benchmark to compare individual patient test results and therefore play a central role in evaluation of laboratory results. Reliable and accurate RI are critical for accurate interpretation of laboratory results. Reference intervals should be established using a large number of samples from healthy volunteers tiered for covariates including age, gender and ethnicity. Homovanillic acid and VMA excretion is known to increase with age while creatinine excretion increases with muscle mass. Hence, it is critical to correct both analytes’ values for creatinine excretion. No differences have been reported in HVA and VMA concentration in relation to gender. Hence, age-related reference intervals and decision limits must take into account these variations. It is recommended that each laboratory maintain local RI related to the observed population (*67*). For the adult population, it is relatively easier to collect blood or urine samples. This approach, however, is difficult in the pediatric population due to limitations in obtaining samples from healthy participants. An alternative is to utilize a large number of laboratory results and analyze them with specific statistical methods (*68*).

Diagnosis of catecholamine producing pediatric tumors has been historically assessed in urine, hence RI for this matrix is most studied. Although 24-hour collection of urine is preferred, this becomes difficult for children. Hence, spot urine collection is preferred with correction using excreted creatinine concentrations. This, however, introduces a new variable as creatinine output varies with diet, exercise and muscle mass (*69*). Data from published studies are compiled into Table 2 and 3 depending on units of reference ranges, as shown below. The reference ranges vary based on the analytical testing method used, population size and selection method.

**Table 2:** Reference ranges for HVA and VMA in mmol/mol creatinine

**Reference and method used**	**Sample size (N)**	**Age group**	**HVA** **(mmol/mol Cr)**	**VMA (mmol/mol Cr)**
	926	All	< 13.97	< 7.82
Barco, 2014 (*68*) HPLC-ECD		All < 18 months	< 16.46	< 11.48
		All > 18 months	< 9.87	< 5.54
	*Healthy = 314; With suspected NET = 36	< 1 yr		< 20.27
		1 yr		< 17.7
Tohmola, 2015 (*70*) LC-MS/MS		2-4 yrs		< 9.48
		5-9 yrs		< 8.68
		10-15 yrs		< 6.74
	1658	Under 1 yr	< 23.1	< 12.9
		1 or 2 yrs	< 18	< 12.3
		3 or 4 yrs	< 12	< 8.9
Davidson, 2011 (*71*) HPLC-ECD		5 to 7 yrs	< 10.5	< 7.9
		8 to 10 yrs	< 9.8	< 5
		11 to 13 yrs	< 5.9	< 5.6
		14 to 19 yrs	< 5.0	< 5.5
	367	<1 yrs	< 16.8	< 13
Kellie, 1986 (*72*) HVA: on-column gas chromatography		1-5 yrs	< 13.8	< 7.7
VMA: Method by Pisano *et al.* extraction and spectrophotometric determination		5-10 yrs	< 11	< 5.3
		10-15 yrs	< 7	> 4.1
	12	< 3 months	< 21.7	
	28	3 - 12 months	< 27.9	
Tuchman, 1985 (*73*) capillary GC	15	1 - 2 yrs	< 19.7	
	22	2 - 5 yrs	< 19.9	
	21	5 - 10 yrs	< 14.7	
	13	10 - 15 yrs	< 8.4	
	10	0 - 1 months	< 17.6	
	12	1 - 4 months	< 13.2	
Ito, 1985 (*74*) HPLC	10	4 - 7 months	< 8.3	
	10	7 - 10 months	< 5	
	9	10 - 16 months	< 3.4	
	35	3-6 months	< 9.63	< 6.17
Premel- cabic, 1986 (*75*) HPLC	17	6-10 months	< 7.14	< 4.28
	24	10 - 16 months	< 6.4	< 5.02
	181 for HVA 163 for VMA	<1 months (N=38)	< 16.8	< 13
		1-5 months (N=67)	< 13.8	< 7.7
Kellie, 1986 (*72*) GC and spectrophotometry		5-10 months (N=45)	< 11	< 5.3
		10-15 months (N=31)	< 7	< 4.1
	140	≤ 14 months	<1 months to 26	
			2 months to 18	
Henderson, 1992 (*76*) capillary GC			4 months to 15	
			6 months to 14.5	
			10 months to 14	
			14 months to 12	
Mathieu, 1996 (*77*) HPLC-ECD	105,293	4 months - 1 yr	< 20.56	< 13.82
Craft, 1989 (*78*) GC with flame ionization detection and GC-MS	1st phase: 95	6 months	< 25.15	< 16.27
	2nd phase: 2304	6 months	< 24.22	< 14.28
Parker, 1992 (*79*) GC and Flame ionization detection	20,829	6 months	< 24	< 15
Cole,1995 (*80*) GC-MS	10,000	6 months	< 24.2	< 14.7
	33	0 - 12 m	< 23.99	< 13.67
	44	1 yrs	< 23.18	< 14.5
Shen, 2019 (*81*) LC-MS/MS	125	2 - 4 yrs	< 45.42	< 10.58
	339	5 - 9 yrs	< 43.13	< 7.61
	195	10 - 14 yrs	< 21.16	< 6
	39	15 - 18 yrs	< 16.18	< 5.99
	57	<1 yrs	< 25	
	43	1<3 yrs	< 15	
Fitzbibbon, 1992 (*82*) HPLC-ECD	46	>3<5 yrs	< 15	
	45	>5<8 yrs	< 14	
	41	>8<11 yrs	< 10	
	51	>11-14 yrs	< 5	
Cr – creatinine. VMA – vanillylmandelic acid. HVA- homovanillic acid. HPLC-ECD - high performance liquid chromatography coupled with electrochemical detection. LC-MS/MS – liquid chromatography tandem mass spectrometry. HPLC - high performance liquid chromatography. GC-Gas chromatography. LC-Liquid chromatography. MS-mass spectrometry. m-month. yrs-years. *The authors did not differentiate between healthy individuals and suspected NET, with regards to total N (sample population). 1^st^ phase indicates “pilot study” and 2nd phase indicates “clinical trial”.				

**Table 3:** Reference ranges for HVA and VMA in µmol/24 hours

**Reference and method used**	**Sample Size (N)**	**Age group**	**HVA** **(µmol/24 hrs)**	**VMA** **(µmol/24 hrs)**
	35	3-6 months	< 23.607	< 13.12
Premel- Cabic, 1986 (*75*) HPLC	17	6-10 months	< 25.803	< 16.15
	24	10-16 months	< 47.763	< 26.24
De Schaepdryver, 1978 (*83*) HPLC	17	0-6 months	< 4.94	/
Nishi, 1986 (*84*) HPLC	24	6-11 months	< 12.41	< 8.78
VMA - vanillylmandelic acid. HVA- homovanillic acid. HPLC - high performance liquid chromatography.				

## Laboratory interpretation and follow-up

Potential impact of analytical and preanalytical interferences needs to be considered by clinicians and laboratories providing the tests, for appropriate interpretation of results. Accurate interpretation depends on patient presentation, symptoms and medical history, while also depending on the patient’s population and background. Patients being tested due to non-specific symptoms such as hypertension, sweating and headache could arise from other conditions like primary hypertension and migraine. In this patient group, while specificity and positive predictive value are limited, the diagnostic sensitivity and negative predictive value are relatively high (*85*). Measurement of O-methylated catecholamine metabolites can provide insights into location, size, type of disease mutation and aggressiveness of the disease, by assessing the patterns of increase of these metabolites above the reference intervals (*86*). Neuroblastoma can be ruled in or out depending on repeated measurements of VMA/HVA concentration along with clinical findings, abdominal mass symptoms and magnetic resonance imaging (MRI) findings to decide if a biopsy is necessary (*10*). However, a small percentage of NBL tumors are non-secretory, hence, repeated measurements will not increase detection rate in these cases (*87*).

Catecholamines and metabolites are not helpful in detection of relapse for patients who have previously been treated for NBL. In these cases, local recurrence or progression of the disease was established by ultrasound, computed tomography or magnetic resonance imaging (*88*).

## Future perspectives

There is large variation in reporting of test results from different laboratories. This variability could arise from several factors including sample collection, inadequate sample sizes, age groups of the population and the statistical methods used. Recent studies use graphical representation of data identifying outliers and complementing statistical analysis. Although quantifying urine HVA and VMA aids in diagnosis and follow up of NBL, these tests are not 100% specific or sensitive. This could also signify that neuroblastoma is a heterogeneous disease.

Early detection of NBL may provide improved survival chances. This premise led to several mass-screening programs; however, the results have not been promising. Although the screening led to increased cases of diagnosis, a decrease in mortality rates was not observed. This could be due to the high tendency of spontaneous regression or due to difficulty in the screening system used (*88*). The difference in pattern of neuroblastoma growth also arises from the stage of the disease and hence factors such as analytical system used, age of the population and repeated sampling should be considered (*89*). While HVA and VMA are the foundational biochemical markers for diagnosis and risk stratification of NBL, their prognostic utility is limited by heterogeneity and variable secretion across tumor subtypes. This underscores the need for complementary biomarkers that capture tumor burden, metabolic programming, oxidative stress and microenvironmental interactions (*19*, *90*).

The predictive power and detection accuracy of HVA and VMA could be substantially improved through combination with emerging metabolites such as vanillactic acid (VLA), 3-methoxytyramine sulfate (MTS), lactate dehydrogenase (LDH) neuron-specific enolase (NSE), and CA125 (*90*). Serum LDH and NSE reflect proliferative activity and neuroendocrine differentiation (*91*, *92*). Further, CA125 has emerged as a potential indicator of tumor-host interactions in high risk NBL (*93*, *94*). Alterations in lipid metabolism, including dysregulated very-long-chain fatty acids (VLCFA) profiles, are increasingly recognized as hallmarks of pediatric tumors offering novel prognostic insights when combined with catecholamine metabolism (*94*). High LDH values positively correlated with a worse prognosis (*95*).

Future efforts should prioritize multivariate modeling approaches - including machine-learning-based composite scores - to validate multi-marker panels that outperform single-analyte testing for risk stratification, early relapse detection, and response assessment. Prospective multicenter validation and integration with clinical and molecular risk factors will be essential to enable clinical translation of these combined metabolite signatures into precision-based NBL care (*89*, *96*).

Variability can be reduced through improved assays, increased standardization of sample preparation, methods and values, using harmonized reference intervals and establishing local reference ranges with appropriate healthy age groups. Use of age-adjusted reference intervals could circumvent variability arising from differences in age of population groups. The reference ranges should be established according to the International Federation of Clinical Chemistry (IFCC) recommendations. Although the incidence of NBL is very low, mass screening of infants for the disease should be established at different time intervals to enable improved prognosis.

In conclusion, NBL remains a clinically heterogenous pediatric malignancy in which early detection is critical to improving outcomes. Urinary catecholamine metabolites, HVA and VMA, play central role in the biochemical evaluation of this disease. The high specificity and sensitivity across age groups make them valuable biomarkers for diagnosis and monitoring NBL.

This review highlights that accurate interpretation of HVA and VMA is dependent on age-specific reference intervals, normalization to creatinine and consideration of pre-analytical variables such as specimen type, collection method and sample stability. While 24-hour urine collections have traditionally been recommended, increasing evidence supports the clinical utility and practicality of spot urine samples in pediatric populations.

Advances in analytical methodologies have further improved the reliability and precision of HVA and VMA measurements, especially LC-MS/MS, which is virtually free of analytical interferences. Overall, integrating robust analytical practices with clinically appropriate interpretation of urinary HVA and VMA supports their use as key biomarkers in the management of NBL.

## Data Availability

No data was generated since this is a literature review manuscript.
